# Survey of Natural Language Processing Techniques in Bioinformatics

**DOI:** 10.1155/2015/674296

**Published:** 2015-10-07

**Authors:** Zhiqiang Zeng, Hua Shi, Yun Wu, Zhiling Hong

**Affiliations:** ^1^College of Computer and Information Engineering, Xiamen University of Technology, Xiamen 361024, China; ^2^Software School, Xiamen University, Xiamen 361005, China

## Abstract

Informatics methods, such as text mining and natural language processing, are always involved in bioinformatics research. In this study, we discuss text mining and natural language processing methods in bioinformatics from two perspectives. First, we aim to search for knowledge on biology, retrieve references using text mining methods, and reconstruct databases. For example, protein-protein interactions and gene-disease relationship can be mined from PubMed. Then, we analyze the applications of text mining and natural language processing techniques in bioinformatics, including predicting protein structure and function, detecting noncoding RNA. Finally, numerous methods and applications, as well as their contributions to bioinformatics, are discussed for future use by text mining and natural language processing researchers.

## 1. Introduction

Text mining and natural language processing refer to comprehending and analyzing natural language by using computer algorithms and programs. It is an important research direction in the application field of artificial intelligence. Research on natural language processing and text mining has been reported as early as the emergence of computers. With continuous and extensive research on machine learning and data mining algorithms, existing text mining technologies have achieved good results in automatic abstraction, automatic question answering, web relational network analysis, and anaphora resolution [1, 2].

Bioinformatics is an interdiscipline that emerged with the progress and accomplishment of the Human Genome Project. It predicts and solves live science problems related to genetics by using computer and statistical informatics. Data storage, retrieval, and analysis are the key processes in bioinformatics [3–7]. The National Center for Biotechnology Information established various databases for biological data, including sequence databases for storing DNA and protein data (e.g., dbEST and dbSNP) [8, 9], Online Mendelian Inheritance in Man database for storing disease data, Gene Expression Omnibus database for storing gene chip data, and PubMed database for storing biological and medical literature [10].

**Figure 1:**
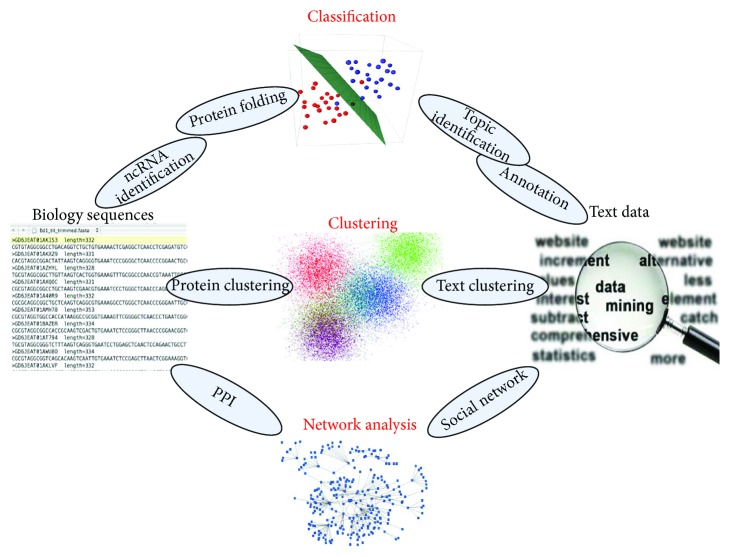
Problems and methodology relationship between NLP and bioinformatics.

Text mining and natural language processing techniques are necessary to retrieve user preference knowledge from expanding databases. Therefore, researchers retrieve papers on certain topics of interest, such as determining protein-protein interactions, from PubMed using computer algorithms and programs. With the cracking of genetic codes, researchers have determined that biological sequences, particularly protein sequences, are similar to human language in terms of composition. In addition to using text mining to retrieve bioinformatics articles directly, an increasing number of researchers are regarding protein sequences as a special “text” and analyzing them based on existing text mining technologies. The relationship between bioinformatics and natural language processing is shown in Figure 1. Researchers have also predicted the structures and functions of proteins. Based on these two aspects, we summarize the text mining technologies used in bioinformatics research. We aim to present these technologies to more bioinformatics researchers and hope that the number of researchers who can use good text mining technologies in bioinformatics studies will increase.

## 2. Mining Bioinformatics Literature

The development of text mining technology plays an important role in retrieving biological literature, particularly in establishing biological information databases. A special workshop on biological literature retrieval problems was conducted during the Annual Meeting of the Association for Computational Linguistics and the Annual International Conference on Intelligent Systems for Molecular Biology in 2005 to discuss literature mining problems related to bioinformatics. Extracting protein-protein interactions and the relationship between gene functions and diseases are two leading application subjects.

### 2.1. Extracting Protein-Protein Interactions

Extracting the protein interaction network is an important research topic in bioinformatics and systems biology [11–14]. In previous studies, researchers searched for protein-protein interactions manually. However, with the exponential growth of biological literature, a program that can recognize protein-protein interactions automatically from PubMed abstracts is necessary. Nevertheless, no unified naming rule for proteins has been established yet. Many proteins and genes use the same name. Consequently, recognizing protein names from the literature abstracts and further determining their interactions are key problems in the application of text mining in searching for protein-protein interactions.

Initially, researchers extracted protein-protein interactions through statistical and counting methods. They manually created dictionaries of protein names and then searched abstracts that involve elements occurring at least twice. On this basis, researchers determined that associated proteins interact with one another [15]. Some researchers also used dynamic planning to extract and compare protein-protein interactions [16].

Extracting protein-protein interactions has been a research hot spot in bioinformatics for a long time and has attracted an increasing number of researchers in the fields of text mining and natural language processing. First, the grammar of literature abstracts is analyzed more carefully, rather than making a simple statistics of dictionary words. Kim et al. converted a complicated semantic structure analysis into calculating the shortest path in a graph by creating a nucleus [17]. Similar analysis methods of literature abstracts include grammatical analysis [18–21], context-free grammar analysis [22], ontology analysis [23], and other information retrieval methods. Protein-protein interactions are examined using these analysis methods. In addition, many machine learning methods, such as ensemble learning [24] and Bayesian network [25], are applied to recognize protein names and interactions.

### 2.2. Extracting the Relationship between Gene Functions and Diseases

Extracting protein-protein interactions involves searching for two proteins in the text and determining whether they interact with each other. Similarly, extracting the relationship between gene functions and diseases also involves searching for gene names and disease names simultaneously in the literature and then determining whether a particular gene is related to a certain disease [26].

In general, such extraction process can be divided into three steps. First, the abstracts of associated papers are searched through comparison with a dictionary. Second, the search scope has to be expanded forward and backward sometimes based on the location of the related word or clause to ensure accuracy. Finally, facts are evaluated using grammar analysis methods or machine learning methods. Such extraction methods frequently yield good results for special genes and diseases. Bui et al. examined the relationship between drugs and HIV variation in PubMed [27]. Jiang et al. determined the relationship between approximately 3000 microRNAs and different diseases based on the naming rule of microRNA [28]. Cheng et al. developed a text mining system based on the relationship among human diseases, variations, and drug effects [29]. Iossifov et al. focused on investigating malformations of human and mouse encephalon [30]. Jensen et al. made a detailed summary of related document databases, literature mining software, and functions [31].

### 2.3. Retrieving References

A considerable amount of bioscience literature has been published. Searching for interacting proteins and examining the relationship between genes and diseases are only two application cases. Text mining technology is required to obtain answers to many other bioscience and bioinformatics problems in various databases, such as PubMed.

Biological literature mining and related problem solving have to cope with two major problems, namely, recognizing name entities and extracting relations. These problems are mainly solved by (1) methods based on linguistic analysis [32], (2) methods based on dictionaries [33], (3) machine learning methods [34, 35], and (4) statistical methods [36].

Several important databases are also selected with text mining. STRING [37] and BioGRID [38] are built for protein-protein interaction with literature mining. For predicting gene function, PubTator [39] and GeneCards [40] are important databases using text mining techniques. Related works were reviewed in detail in Huang and Lu's work [41] recently. As the development of crowdsource, artificial text searching and mining can also be helpful for biomedicine literature collection [42].

Moreover, converting PubMed database into an Extensible Markup Language relational database [43] and a fuzzy search of papers and author names through short-term matching are also current research hot spots [44].

## 3. Applying Text Mining Technologies to Protein Research

DNA and protein sequences are a meaningful genetic language and are regarded as the sealed book of life. Therefore, an increasing number of natural language processing and text mining algorithms are being applied to study bioinformatics. For example, latent semantic analysis was applied to protein remote homology detection [45, 46], and protein spectral analysis originates from word frequency statistics in natural language processing. Furthermore, some grammar rules of protein, DNA, and RNA sequences were discovered, and several web servers were constructed so as to extract these features and rules [47].

### 3.1. Predicting Protein Structure

Protein structure determines function [48]. Hence, it should be analyzed to determine protein function. The structural analysis of protein mainly focuses on certain protein sequences and classifies regions into the *α*-helix, *β*-lamella, and protein disordered regions. Predicting the *α*-helix and *β*-lamella regions is the same as predicting the secondary protein structure.

If a protein sequence is regarded as a natural language, then analyzing the type of protein in a region is similar to calibrating grammar in natural language processing. First, the secondary protein structure is predicted by combining rules and statistics [49–52]. However, faced with the bottleneck of statistical prediction, some researchers have proposed using machine learning prediction methods, including methods based on artificial neural network (ANN) [53], support vector machine (SVM) [54, 55], random forest [56–58], and maximum entropy [59].

Predicting the protein disordered region is also conducted. This region refers to the area without a stable or unique 3D structure in the protein space structure. Many text mining and machine learning methods, including ANN [60–62], SVM [63–65], conditional random field [66], and random forest [67], have been used to predict the protein disordered region. Common existing server addresses are listed in Table 1.

### 3.2. Predicting Protein Function

Predicting protein function is one of the most basic research topics in bioinformatics. It involves predicting protein-protein interactions and interaction sites [68, 69], localizing subcellular protein [70–78], predicting and classifying transmembrane protein [79–82], protein remote homology detection [83, 84], classifying protein functions [85–93], recognizing multifunctional enzymes [94–96], and DNA binding protein identification [97, 98].

The protein sequence is easy to determine. Similar to natural language, the protein sequence has many complicated rules. However, summarizing and understanding the rules of protein sequences are difficult. Therefore, analyzing and predicting the “protein language” expressed by amino acid sequences by using computational linguistics and machine learning methods are necessary. Through these procedures, we may be able to understand the functions of protein sequences.

Predicting protein-protein interactions is one of the most basic research topics in protein functions. Many researchers are committed to predicting whether two protein sequences exhibit interactions. To date, many machine learning methods have been applied, including SVM [99], kernel method [100, 101], decision-making tree [102, 103], random forest [104], Bayesian network [105], and the autoregressive model [106]. Several text processing methods, such as ontology annotation and sample weighting [107], are used to detect features and process training data. When predicting protein-protein interactions, researchers also aim to analyze the region of protein-protein interactions, which is used to predict protein-protein interaction sites. Information approaches commonly used in grammatical analyses, such as condition random fields [108] and a hidden Markov model (HMM) [109], have been used to analyze interaction sites and have achieved good results. Moreover, random forest [110], SVM [111], ANN [112], Bayesian network [113], linear regression [114], and other machine learning methods are used to predict protein-protein interaction sites. Nevertheless, some researchers doubt that determining the protein sequence alone is inadequate to provide sufficient information for predicting interactions [115]. Text mining and machine learning researchers should develop new features and classification methods to solve this problem. The websites of existing common software used to predict protein-protein interactions and interaction sites are provided in Table 2.

## 4. Applying Natural Language Processing Techniques to Noncoding RNA Identification

### 4.1. Comparative RNA Prediction Methods

Alignment is also an important topic in natural language processing. DNA or RNA sequences can also be viewed as text. Sequence-based multiple sequence alignment methods can be used only at the sequence similarity level. The secondary structures of ncRNAs are usually more conserved than their sequences [116, 117]; for example, miRNA precursors share the common hairpin-like structure and tRNAs form cloverleaf structures [118, 119]. The functions of many ncRNAs are therefore determined by their secondary structure rather than by their sequences. As a result, structure-based multiple sequence alignment methods have been developed to align an input sequence to known ncRNA structures to determine the ncRNA class to which the input sequence belongs.

LocARNA [120] can produce fast and high-quality pairwise and multiple alignments of RNA sequences. It uses a complex RNA energy model for simultaneous folding and sequence/structure alignment of the RNAs. LocARNA performs global and local sequence alignments as well as local structural alignment of RNA molecules. An upgraded version of LocARNA, called LocARNA-P, has been developed recently [121]. The new version incorporates a probabilistic model that can compute accurate multiple alignments based on a probabilistic consistency transformation and reliability profiles for assessing local alignment quality and localizing RNA motifs. These features are based on computing sequence and structure match probabilities based on the LocARNA alignment model.

Although comparative methods perform well in most cases, they have three intrinsic limitations: (1) they are highly dependent on the availability of homologous sequences or structures and cannot make predictions when no relevant sequence similarity or structure similarity is available; (2) they cannot correctly identify real ncRNAs that have low homology with known ncRNAs; and (3) they can identify only ncRNAs that are homologous with members of known ncRNA classes but cannot identify members of novel ncRNA classes. Most lncRNAs (long noncoding RNAs) cannot be predicted using comparative methods because they do not have specific structures or sequence similarity. These limitations mean that comparative methods display low specificity for identifying ncRNAs. The multiple sequence alignment tools that are currently available are listed in Table 3.

### 4.2. Noncomparative RNA Prediction Methods

The noncomparative methods are independent of homologous information and can, therefore, detect nonconserved ncRNAs. Most noncomparative methods employ machine learning techniques to make the predictions [122], which are similar to the text mining techniques.

Because of the importance of RNA structure, several computational RNA folding tools have been developed, such as mfold, RNAfold, vsfold, evofold, and sfold. Generally, these algorithms determine the folded secondary structure from and input sequence by optimizing the intermolecular base pairing to minimize the free energy. Some miRNA identification methods are shown in Table 4 and existing RNA secondary prediction tools are listed in Table 5.

## 5. Conclusion and Future Research

As research on natural language and text mining methods develops, different application fields will be the key to future studies. Interdisciplines represented by bioinformatics are becoming the focus of an increasing number of information science researchers. The application of text mining technologies and methods in bioinformatics study will become the focus of text mining researchers. Meanwhile, bioinformatics researchers have to learn text mining technologies intensively to solve specific bioinformatics problems.

In retrieving biological literature, apart from the aforementioned prediction of protein-protein interactions and gene-disease relationship, many problems, particularly those that require updating literature retrieval results, such as the relationships between adverse drug reaction and molecule composition as well as among single nucleotide polymorphism sites, diseases, and adverse drug effects, require the use of text mining to search for related knowledge in a literature database.

In bioinformatics, nearly all studies related to proteomics and predicting protein structure according to amino acid sequences can be conducted using text mining and natural language processing technology. Many mature texts mining technologies, such as word frequency statistics, condition random fields, HMM, and context-free grammar, have been successfully applied to predict secondary protein structures, irregular regions, interactions, and interaction sites. However, the latest research results in text mining and natural language processing should be verified by applying them in protein and DNA languages. No effective computation method is available yet for predicting third and fourth protein structures, protein homology remote detection, protein disordered region detection, interaction network establishment, and drug target prediction. Information science researchers should develop and provide more effective algorithms. In addition, new machine learning and text mining methods (e.g., semisupervised learning and active learning) have been proposed and will be applied in biological literature retrieval and bioinformatics. At present, recommending systems based on feedback has become a new hot spot problem in retrieving biological literature. And the Hadoop technique for big data is another hot spot for biology sequences [123].

The development of bioinformatics relies on information science. In particular, text mining and natural language processing researchers should provide a more extensive application space. Researchers of text mining algorithms should develop more effective intelligent algorithms based on the characteristics of biological data. This study does not only summarize text mining methods used in bioinformatics and corresponding problems, but it also provides related websites of successful prediction software. Recently, text mining researchers who are involved in bioinformatics can test and compare different types of software. The authors hope that the number of text mining researchers who can apply their own methods in bioinformatics will increase, which will facilitate the development of bioinformatics and even genetic studies.

## Figures and Tables

**Table 1:** Web server for protein disorder prediction.

Problem	Name	Websites	Input format
Protein disorder prediction	DisProt	http://www.disprot.org/pondr-fit.php	Fasta or EMBL sequence format
		http://www.disprot.org/metapredictor.php	
		http://www.dabi.temple.edu/disprot/predictor.php	
	DisEMBL	http://dis.embl.de/	SwissProt ID
	DRIPPRED	http://www.sbc.su.se/~maccallr/disorder/cgi-bin/submit.cgi	Only plain sequence; one sequence once; slow
	FoldIndex	http://bip.weizmann.ac.il/fldbin/findex	Only plain sequence; one sequence once
	IUPred	http://iupred.enzim.hu/	SwissProt ID or plain sequence
	PONDR	http://www.pondr.com/cgi-bin/PONDR/pondr.cgi	Fasta
	PSIPRED	http://bioinf.cs.ucl.ac.uk/psipred/?disopred=1	Raw sequence or fasta format
	SCRATCH	http://scratch.proteomics.ics.uci.edu/	Only plain sequence; one sequence once; slow
	Spritz	http://distill.ucd.ie/spritz/	Raw sequence or fasta format
	RONN	http://www.strubi.ox.ac.uk/RONN/	Fasta, but only one sequence once

**Table 2:** Web server for protein-protein interaction and sites prediction.

Problem	Name	Websites	Input format
Protein interaction sites prediction	PPISP	http://pipe.scs.fsu.edu/ppisp.html	PDB file
		http://pipe.scs.fsu.edu/meta-ppisp.html	
	Protemot	http://protemot.csbb.ntu.edu.tw/index.html	PDB ID
	SPPIDER	http://sppider.cchmc.org	PDB file or PDB ID
	Whiscy	http://nmr.chem.uu.nl/Software/whiscy/index.html	PDB file

Protein-protein interaction prediction	InterPreTS	http://www.russell.embl.de/cgi-bin/tools/interprets.pl	Fasta, 40 sequences at most
	PIE	http://www.ncbi.nlm.nih.gov/CBBresearch/Wilbur/IRET/PIE/	Gene ID or name
	PPI	http://121.192.180.204:8080/PPI/Home.jsp	Fasta
	PredHS	http://www.predhs.org/	PDB files, 10 files at most
	Pred-PPI	http://cic.scu.edu.cn/bioinformatics/predict_ppi/default.html	Two fasta sequences
	Prism	http://cosbi.ku.edu.tr/prism/	Two PDB IDs or PDB files
	Struct2Net	http://groups.csail.mit.edu/cb/struct2net/webserver/	Gene names or keywords

**Table 3:** Multiple sequence alignment tools.

Tool	Alignment method	URL
BLAT	Sequence-based	http://genome.ucsc.edu/
BLAST		http://www.ncbi.nlm.nih.gov/
BWA-SW		http://bio-bwa.sourceforge.net

Multilign	Structure-based	http://rna.urmc.rochester.edu/
FoldalignM		http://foldalign.ku.dk/
LocARNA/LocARNA-P		http://www.bioinf.uni-freiburg.de/Software/LocARNA/
MASTR		http://mastr.binf.ku.dk/
RAF		http://contra.stanford.edu/contrafold/
RNASampler		http://ural.wustl.edu/software.html
RNAshapes		http://bibiserv.techfak.uni-bielefeld.de/rnashapes/
RNAalifold		http://www.tbi.univie.ac.at/RNA/
StemLoc		N.A.
MAFFT		http://mafft.cbrc.jp/alignment/software/index.html
MiRAlign		http://bioinfo.au.tsinghua.edu.cn/miralign/

**Table 4:** miRNA identification methods.

Method	URL	Online service	Local service
MiPred	http://www.bioinf.seu.edu.cn/miRNA/	✓	✓
microPred	http://www.cs.ox.ac.uk/people/manohara.rukshan.batuwita/microPred.htm		✓
TripletSVM	http://bioinfo.au.tsinghua.edu.cn/mirnasvm		✓
PlantMiRNAPred	http://nclab.hit.edu.cn/PlantMiRNAPred/	✓	✓
miRNApre	http://121.192.180.205:8080/miRNApreWeb/	✓	✓
MIReNA	http://www.ihes.fr/~carbone/data8/		✓
HuntMi	http://adaa.polsl.pl/agudys/huntmi/huntmi.htm		✓
Mirident	http://www.regulatoryrna.org/pub/mirident		✓
CSHMM	http://web.iitd.ac.in/~sumeet/mirna/		✓
HeteroMirPred	http://ncrna-pred.com/premiRNA.html	✓	✓

**Table 5:** Secondary prediction tools.

Tool	URL
RNAfold	http://rna.tbi.univie.ac.at/cgi-bin/RNAfold.cgi
RNAstructure	http://rna.urmc.rochester.edu/rnastructure.html
mfold	http://www.bioinfo.rpi.edu/applications/mfold/
vsfold	http://www.rna.it-chiba.ac.jp/~vsfold/vsfold4/
evofold	http://users.soe.ucsc.edu/~jsp/EvoFold/
sfold	http://sfold.wadsworth.org/cgi-bin/index.pl

## References

[1] Lin C., Huang Z., Yang F., Zou Q. (2012). Identify content quality in online social networks. *IET Communications*.

[2] Chen L., Chun L., Ziyu L., Quan Z. (2013). Hybrid pseudo-relevance feedback for microblog retrieval. *Journal of Information Science*.

[3] Li Y., Wang C., Miao Z. (2015). ViRBase: a resource for virus-host ncRNA-associated interactions. *Nucleic Acids Research*.

[4] Wang L., Qian K., Huang Y. (2015). SynBioLGDB: a resource for experimentally validated logic gates in synthetic biology. *Scientific Reports*.

[5] Wang Y., Chen L., Chen B. (2013). Mammalian ncRNA-disease repository: a global view of ncRNA-mediated disease network. *Cell Death & Disease*.

[6] Zhang X., Wu D., Chen L. (2014). RAID: a comprehensive resource for human RNA-associated (RNA-RNA/RNA-protein) interaction. *RNA*.

[7] Li Y., Zhuang L., Wang Y. (2013). Connect the dots: a systems level approach for analyzing the miRNA-mediated cell death network. *Autophagy*.

[8] Wang J., Zou Q., Guo M. Z. (2010). Mining SNPs from EST sequences using filters and ensemble classifiers. *Genetics and Molecular Research*.

[9] Wang J., Zhang L., Zou Q., Tan J., Chen X., Wu Y. (2014). Association studies on mtDNA and Parkinson’s disease population discrimination using the statistical classification. *Current Bioinformatics*.

[10] Zou Q., Li J., Hong Q. Prediction of microRNA-disease associations based on social network analysis methods.

[11] Liu B., Wang X., Lin L., Tang B., Dong Q., Wang X. (2009). Prediction of protein binding sites in protein structures using hidden Markov support vector machine. *BMC Bioinformatics*.

[12] Guo F., Li S. C., Du P., Wang L. (2014). Probabilistic models for capturing more physicochemical properties on protein-protein interface. *Journal of Chemical Information and Modeling*.

[13] Guo F., Li S. C., Wang L., Zhu D. (2012). Protein-protein binding site identification by enumerating the configurations. *BMC Bioinformatics*.

[14] Guo F., Li S. C., Wang L. (2011). Protein-protein binding sites prediction by 3D structural similarities. *Journal of Chemical Information and Modeling*.

[15] Huang M., Zhu X., Hao Y., Payan D. G., Qu K., Li M. (2004). Discovering patterns to extract protein-protein interactions from full texts. *Bioinformatics*.

[16] Hao Y., Zhu X., Huang M., Li M. (2005). Discovering patterns to extract protein-protein interactions from the literature: part II. *Bioinformatics*.

[17] Kim S., Yoon J., Yang J. (2008). Kernel approaches for genic interaction extraction. *Bioinformatics*.

[18] Ono T., Hishigaki H., Tanigami A., Takagi T. (2001). Automated extraction of information on protein-protein interactions from the biological literature. *Bioinformatics*.

[19] Fundel K., Küffner R., Zimmer R. (2007). RelEx—relation extraction using dependency parse trees. *Bioinformatics*.

[20] Šarić J., Jensen L. J., Ouzounova R., Rojas I., Bork P. (2006). Extraction of regulatory gene/protein networks from Medline. *Bioinformatics*.

[21] Friedman C., Kra P., Yu H., Krauthammer M., Rzhetsky A. (2001). GENIES: a natural-language processing system for the extraction of molecular pathways from journal articles. *Bioinformatics*.

[22] Temkin J. M., Gilder M. R. (2003). Extraction of protein interaction information from unstructured text using a context-free grammar. *Bioinformatics*.

[23] Skusa A., Rüegg A., Köhler J. (2005). Extraction of biological interaction networks from scientific literature. *Briefings in Bioinformatics*.

[24] Malik R., Franke L., Siebes A. (2006). Combination of text-mining algorithms increases the performance. *Bioinformatics*.

[25] Chowdhary R., Zhang J., Liu J. S. (2009). Bayesian inference of protein–protein interactions from biological literature. *Bioinformatics*.

[26] Zou Q., Li J., Wang C., Zeng X. (2014). Approaches for recognizing disease genes based on network. *BioMed Research International*.

[27] Bui Q.-C., Nualláin B. T., Boucher C. A., Sloot P. M. A. (2010). Extracting causal relations on HIV drug resistance from literature. *BMC Bioinformatics*.

[28] Jiang Q., Wang Y., Hao Y. (2009). miR2Disease: a manually curated database for microRNA deregulation in human disease. *Nucleic Acids Research*.

[29] Cheng D., Knox C., Young N., Stothard P., Damaraju S., Wishart D. S. (2008). PolySearch: a web-based text mining system for extracting relationships between human diseases, genes, mutations, drugs and metabolites. *Nucleic Acids Research*.

[30] Iossifov I., Rodriguez-Esteban R., Mayzus I., Millen K. J., Rzhetsky A. (2009). Looking at cerebellar malformations through text-mined interactomes of mice and humans. *PLoS Computational Biology*.

[31] Jensen L. J., Saric J., Bork P. (2006). Literature mining for the biologist: from information retrieval to biological discovery. *Nature Reviews Genetics*.

[32] Müller H.-M., Kenny E. E., Sternberg P. W. (2004). Textpresso: an ontology-based information retrieval and extraction system for biological literature. *PLoS Biology*.

[33] Uramoto N., Matsuzawa H., Nagano T., Murakami A., Takeuchi H., Takeda K. (2004). A text-mining system for knowledge discovery from biomedical documents. *IBM Systems Journal*.

[34] Banko M., Cafarella M. J., Soderland S., Broadhead M., Etzioni O. Open information extraction from the web.

[35] Banko M., Etzioni O. The tradeoffs between open and traditional relation extraction.

[36] Abulaish M., Dey L. (2007). Biological relation extraction and query answering from MEDLINE abstracts using ontology-based text mining. *Data and Knowledge Engineering*.

[37] Szklarczyk D., Franceschini A., Wyder S. (2015). STRING v10: protein-protein interaction networks, integrated over the tree of life. *Nucleic Acids Research*.

[38] Chatr-Aryamontri A., Breitkreutz B.-J., Oughtred R. (2015). The BioGRID interaction database: 2015 update. *Nucleic Acids Research*.

[39] Wei C.-H., Kao H.-Y., Lu Z. (2013). PubTator: a web-based text mining tool for assisting biocuration. *Nucleic Acids Research*.

[40] Safran M., Dalah I., Alexander J. (2010). GeneCards version 3: the human gene integrator. *Database*.

[41] Huang C. C., Lu Z. (2015). Community challenges in biomedical text mining over 10 years: success, failure and the future. *Briefings in Bioinformatics*.

[42] Khare R., Good B. M., Leaman R., Su A. I., Lu Z. (2015). Crowdsourcing in biomedicine: challenges and opportunities. *Briefings in Bioinformatics*.

[43] Oliver D. E., Bhalotia G., Schwartz A. S., Altman R. B., Hearst M. A. (2004). Tools for loading MEDLINE into a local relational database. *BMC Bioinformatics*.

[44] Wang J., Cetindil I., Ji S. (2010). Interactive and fuzzy search: a dynamic way to explore MEDLINE. *Bioinformatics*.

[45] Liu B., Wang X., Lin L., Dong Q., Wang X. (2008). A discriminative method for protein remote homology detection and fold recognition combining Top-n-grams and latent semantic analysis. *BMC Bioinformatics*.

[46] Liu B., Xu J., Zou Q., Xu R., Wang X., Chen Q. (2014). Using distances between Top-n-gram and residue pairs for protein remote homology detection. *BMC Bioinformatics*.

[47] Liu B., Liu F., Fang L., Wang X., Chou K. (2015). repDNA: a python package to generate various modes of feature vectors for DNA sequences by incorporating user-defined physicochemical properties and sequence-order effects. *Bioinformatics*.

[48] Liu B., Zhang D., Xu R. (2014). Combining evolutionary information extracted from frequency profiles with sequence-based kernels for protein remote homology detection. *Bioinformatics*.

[49] Chou P. Y., Fasman G. D. (1978). Empirical predictions of protein conformation. *Annual Review of Biochemistry*.

[50] Garnier J., Osguthorpe D. J., Robson B. (1978). Analysis of the accuracy and implications of simple methods for predicting the secondary structure of globular proteins. *Journal of Molecular Biology*.

[51] Dong Q., Wang X., Lin L., Wang Y. (2008). Analysis and prediction of protein local structure based on structure alphabets. *Proteins: Structure, Function and Genetics*.

[52] Dong Q., Wang X., Lin L. (2008). Prediction of protein local structures and folding fragments based on building-block library. *Proteins: Structure, Function and Genetics*.

[53] Rost B., Sander C. (1993). Prediction of protein secondary structure at better than 70% accuracy. *Journal of Molecular Biology*.

[54] Ding H., Lin H., Chen W. (2014). Prediction of protein structural classes based on feature selection technique. *Interdisciplinary Sciences: Computational Life Sciences*.

[55] Lin H., Ding C., Song Q. (2012). The prediction of protein structural class using averaged chemical shifts. *Journal of Biomolecular Structure & Dynamics*.

[56] Lin C., Zou Y., Qin J. (2013). Hierarchical classification of protein folds using a novel ensemble classifier. *PLoS ONE*.

[57] Chen W., Liu X., Huang Y., Jiang Y., Zou Q., Lin C. (2012). Improved method for predicting protein fold patterns with ensemble classifiers. *Genetics and Molecular Research*.

[58] Zhao X., Zou Q., Liu B., Liu X. (2014). Exploratory predicting protein folding model with random forest and hybrid features. *Current Proteomics*.

[59] Liu Y., Carbonell J., Klein-Seetharaman J., Gopalakrishnan V. (2004). Comparison of probabilistic combination methods for protein secondary structure prediction. *Bioinformatics*.

[60] Romero P., Obradovic Z., Li X., Garner E. C., Brown C. J., Dunker A. K. (2001). Sequence complexity of disordered protein. *Proteins: Structure, Function and Genetics*.

[61] Su C.-T., Chen C.-Y., Ou Y.-Y. (2006). Protein disorder prediction by condensed PSSM considering propensity for order or disorder. *BMC Bioinformatics*.

[62] Su C.-T., Chen C.-Y., Hsu C.-M. (2007). IPDA: integrated protein disorder analyzer. *Nucleic Acids Research*.

[63] Ward J. J., Sodhi J. S., McGuffin L. J., Buxton B. F., Jones D. T. (2004). Prediction and functional analysis of native disorder in proteins from the three kingdoms of life. *Journal of Molecular Biology*.

[64] Shimizu K., Hirose S., Noguchi T. (2007). POODLE-S: web application for predicting protein disorder by using physicochemical features and reduced amino acid set of a position-specific scoring matrix. *Bioinformatics*.

[65] Hirose S., Shimizu K., Kanai S., Kuroda Y., Noguchi T. (2007). POODLE-L: a two-level SVM prediction system for reliably predicting long disordered regions. *Bioinformatics*.

[66] Wang L., Sauer U. H. (2008). OnD-CRF: predicting order and disorder in proteins conditional random fields. *Bioinformatics*.

[67] Han P., Zhang X., Norton R. S., Feng Z.-P. (2009). Large-scale prediction of long disordered regions in proteins using random forests. *BMC Bioinformatics*.

[68] Liu B., Wang X., Lin L., Dong Q., Wang X. (2009). Exploiting three kinds of interface propensities to identify protein binding sites. *Computational Biology and Chemistry*.

[69] Liu B., Liu B., Liu F., Wang X. (2014). Protein binding site prediction by combining hidden markov support vector machine and profile-based propensities. *The Scientific World Journal*.

[70] Wang Z., Zou Q., Jiang Y., Ju Y., Zeng X. (2014). Review of protein subcellular localization prediction. *Current Bioinformatics*.

[71] Lin H., Ding H., Guo F.-B., Zhang A.-Y., Huang J. (2008). Predicting subcellular localization of mycobacterial proteins by using Chou's pseudo amino acid composition. *Protein & Peptide Letters*.

[72] Lin H., Ding H., Guo F.-B., Huang J. (2010). Prediction of subcellular location of mycobacterial protein using feature selection techniques. *Molecular Diversity*.

[73] Lin H., Wang H., Ding H., Chen Y.-L., Li Q.-Z. (2009). Prediction of subcellular localization of apoptosis protein using Chou's pseudo amino acid composition. *Acta Biotheoretica*.

[74] Lin H., Chen W., Yuan L.-F., Li Z.-Q., Ding H. (2013). Using over-represented tetrapeptides to predict protein submitochondria locations. *Acta Biotheoretica*.

[75] Ding H., Guo S.-H., Deng E.-Z. (2013). Prediction of Golgi-resident protein types by using feature selection technique. *Chemometrics and Intelligent Laboratory Systems*.

[76] Lin H., Ding C., Yuan L.-F. (2013). Predicting subchloroplast locations of proteins based on the general form of Chou's pseudo amino acid composition: approached from optimal tripeptide composition. *International Journal of Biomathematics*.

[77] Zhu P.-P., Li W.-C., Zhong Z.-J. (2015). Predicting the subcellular localization of mycobacterial proteins by incorporating the optimal tripeptides into the general form of pseudo amino acid composition. *Molecular BioSystems*.

[78] Ding H., Liu L., Guo F.-B., Huang J., Lin H. (2011). Identify golgi protein types with modified mahalanobis discriminant algorithm and pseudo amino acid composition. *Protein & Peptide Letters*.

[79] Zou Q., Li X., Jiang Y., Zhao Y., Wang G. (2013). BinMemPredict: a web server and software for predicting membrane protein types. *Current Proteomics*.

[80] Lin H. (2008). The modified Mahalanobis discriminant for predicting outer membrane proteins by using Chou's pseudo amino acid composition. *Journal of Theoretical Biology*.

[81] Ding C., Yuan L.-F., Guo S.-H., Lin H., Chen W. (2012). Identification of mycobacterial membrane proteins and their types using over-represented tripeptide compositions. *Journal of Proteomics*.

[82] Lin H., Ding H. (2011). Predicting ion channels and their types by the dipeptide mode of pseudo amino acid composition. *Journal of Theoretical Biology*.

[83] Liu B., Wang X., Zou Q., Dong Q., Chen Q. (2013). Protein remote homology detection by combining Chou's pseudo amino acid composition and profile-based protein representation. *Molecular Informatics*.

[84] Liu B., Wang X., Chen Q., Dong Q., Lan X. (2012). Using amino acid physicochemical distance transformation for fast protein remote homology detection. *PLoS ONE*.

[85] Yu G., Rangwala H., Domeniconi C., Zhang G., Yu Z. (2014). Protein function prediction with incomplete annotations. *IEEE/ACM Transactions on Computational Biology and Bioinformatics*.

[86] Zou Q., Wang Z., Guan X., Liu B., Wu Y., Lin Z. (2013). An approach for identifying cytokines based on a novel ensemble classifier. *BioMed Research International*.

[87] Yu G., Rangwala H., Domeniconi C., Zhang G., Yu Z. (2013). Protein function prediction using multi-label ensemble classification. *IEEE/ACM Transactions on Computational Biology and Bioinformatics*.

[88] Ding H., Deng E.-Z., Yuan L.-F. (2014). iCTX-type: a sequence-based predictor for identifying the types of conotoxins in targeting ion channels. *BioMed Research International*.

[89] Liu W.-X., Deng E.-Z., Chen W., Lin H. (2014). Identifying the subfamilies of voltage-gated potassium channels using feature selection technique. *International Journal of Molecular Sciences*.

[90] Ding H., Li D. (2015). Identification of mitochondrial proteins of malaria parasite using analysis of variance. *Amino Acids*.

[91] Ding H., Feng P.-M., Chen W., Lin H. (2014). Identification of bacteriophage virion proteins by the ANOVA feature selection and analysis. *Molecular BioSystems*.

[92] Yuan L.-F., Ding C., Guo S.-H., Ding H., Chen W., Lin H. (2013). Prediction of the types of ion channel-targeted conotoxins based on radial basis function network. *Toxicology in Vitro*.

[93] Lin H., Chen W. (2011). Prediction of thermophilic proteins using feature selection technique. *Journal of Microbiological Methods*.

[94] Cheng X.-Y., Huang W.-J., Hu S.-C. (2012). A global characterization and identification of multifunctional enzymes. *PLoS ONE*.

[95] Lin H., Chen W., Ding H. (2013). AcalPred: a sequence-based tool for discriminating between acidic and alkaline enzymes. *PLoS ONE*.

[96] Zou Q., Chen W., Huang Y., Liu X., Jiang Y. (2013). Identifying multi-functional enzyme by hierarchical multi-label classifier. *Journal of Computational and Theoretical Nanoscience*.

[97] Liu B., Xu J., Fan S., Xu R., Zhou J., Wang X. (2015). PseDNA-Pro: DNA-binding protein identification by combining chou's PseAAC and Physicochemical distance transformation. *Molecular Informatics*.

[98] Liu B., Xu J., Lan X. (2014). IDNA-Prot|dis: identifying DNA-binding proteins by incorporating amino acid distance-pairs and reduced alphabet profile into the general pseudo amino acid composition. *PLoS ONE*.

[99] Bock J. R., Gough D. A. (2001). Predicting protein-protein interactions from primary structure. *Bioinformatics*.

[100] Ben-Hur A., Noble W. S. (2005). Kernel methods for predicting protein-protein interactions. *Bioinformatics*.

[101] Qi Y., Bar-Joseph Z., Klein-Seetharaman J. (2006). Evaluation of different biological data and computational classification methods for use in protein interaction prediction. *Proteins: Structure, Function and Genetics*.

[102] Zhang L. V., Wong S. L., King O. D., Roth F. P. (2004). Predicting co-complexed protein pairs using genomic and proteomic data integration. *BMC Bioinformatics*.

[103] Darnell S. J., Page D., Mitchell J. C. (2007). An automated decision-tree approach to predicting protein interaction hot spots. *Proteins: Structure, Function, and Bioinformatics*.

[104] Chen X.-W., Liu M. (2005). Prediction of protein-protein interactions using random decision forest framework. *Bioinformatics*.

[105] Jansen R., Yu H., Greenbaum D. (2003). A bayesian networks approach for predicting protein-protein interactions from genomic data. *Science*.

[106] Gomez S. M., Noble W. S., Rzhetsky A. (2003). Learning to predict protein-protein interactions from protein sequences. *Bioinformatics*.

[107] Li M.-H., Wang X.-L., Lin L., Liu T. (2006). Effect of example weights on prediction of protein-protein interactions. *Computational Biology and Chemistry*.

[108] Li M.-H., Lin L., Wang X.-L., Liu T. (2007). Protein-protein interaction site prediction based on conditional random fields. *Bioinformatics*.

[109] Friedrich T., Pils B., Dandekar T., Schultz J., Müller T. (2006). Modelling interaction sites in protein domains with interaction profile hidden Markov models. *Bioinformatics*.

[110] Šikić M., Tomić S., Vlahoviček K. (2009). Prediction of protein-protein interaction sites in sequences and 3D structures by random forests. *PLoS Computational Biology*.

[111] Bradford J. R., Westhead D. R. (2005). Improved prediction of protein-protein binding sites using a support vector machines approach. *Bioinformatics*.

[112] Fariselli P., Pazos F., Valencia A., Casadio R. (2002). Prediction of protein-protein interaction sites in heterocomplexes with neural networks. *European Journal of Biochemistry*.

[113] Bradford J. R., Needham C. J., Bulpitt A. J., Westhead D. R. (2006). Insights into protein-protein interfaces using a bayesian network prediction method. *Journal of Molecular Biology*.

[114] Kufareva I., Budagyan L., Raush E., Totrov M., Abagyan R. (2007). PIER: protein interface recognition for structural proteomics. *Proteins*.

[115] Yu J., Guo M., Needham C. J., Huang Y., Cai L., Westhead D. R. (2010). Simple sequence-based kernels do not predict protein-protein interactions. *Bioinformatics*.

[116] Zou Q., Zhao T., Liu Y., Guo M. (2009). Predicting RNA secondary structure based on the class information and Hopfield network. *Computers in Biology and Medicine*.

[117] Zou Q., Lin C., Liu X.-Y., Han Y.-P., Li W.-B., Guo M.-Z. (2011). Novel representation of RNA secondary structure used to improve prediction algorithms. *Genetics and Molecular Research*.

[118] Liu B., Fang L., Liu F. (2015). Identification of real microRNA precursors with a pseudo structure status composition approach. *PLoS ONE*.

[119] Liu B., Fang L., Chen J., Liu F., Wang X. (2015). miRNA-dis: microRNA precursor identification based on distance structure status pairs. *Molecular BioSystems*.

[120] Will S., Reiche K., Hofacker I. L., Stadler P. F., Backofen R. (2007). Inferring noncoding RNA families and classes by means of genome-scale structure-based clustering. *PLoS Computational Biology*.

[121] Will S., Joshi T., Hofacker I. L., Stadler P. F., Backofen R. (2012). LocARNA-P: accurate boundary prediction and improved detection of structural RNAs. *RNA*.

[122] Wang C., Wei L., Guo M., Zou Q. (2013). Computational approaches in detecting non-coding RNA. *Current Genomics*.

[123] Zou Q., Li X.-B., Jiang W.-R., Lin Z.-Y., Li G.-L., Chen K. (2014). Survey of MapReduce frame operation in bioinformatics. *Briefings in Bioinformatics*.

